# Micellar systems of poloxamer 407 and 403 for quercetin delivery

**DOI:** 10.1186/1753-6561-8-S4-P75

**Published:** 2014-10-01

**Authors:** Juscemácia Araujo, Daniele Araujo

**Affiliations:** 1 Universidade Federal do ABC, Santo André, Brazil

## 

The use of poloxamers (PL) have been one of the most widely strategies used for drug delivery systems. The copolymer Poloxamer 407 is a non-ionic surfactant consisting of polioxyethylene (POE) and polioxypropylene (POP) that has many pharmaceutical applications due to its thermoreversible properties. PL are biocompatible and for this reason, are very attractive as carrier systems for administration by different routes, including topical.

This work aimed the development with emphasis on physicohemical characterization of PL hydrogels for quercetin, being a useful strategy to expand the therapeutic application of these compounds as active ingredients in pharmaceutical formulations designed for topical use.^1, 2 ^In the first stage this work was carried out to study the physico-chemical characteristics of quercetin and evelopment of formulations containing PL gels 407 (28% to 30%) and PL 403 (2%) associated to solubilizing agents polyethyleneglycol 600 (PEG 600) and propyleneglycol (PPG).

Systems with higher linearity suggest that PL hydrogels were efficient for permeation across artificial membranes. Besides, thermodynamic profile was obtained from Differential Scanning Calorimetry (DSC) assays, best values of enthalpy (0.580 J / g) and Gibb's free energy (-12245.95 J.K1.mol^-1^) were observed for the system PL 407-PEG 600, showing that PL system present spontaneous micellization, stability and thermorreversibility for quercetin delivery.
